# Preparation and Evaluation of the Biological Properties of Ethanolic Extract of Red Clover: An In Vitro Study

**DOI:** 10.7759/cureus.59762

**Published:** 2024-05-06

**Authors:** Devika Bajpai, Arvina Rajasekar

**Affiliations:** 1 Periodontics, Saveetha Dental College and Hospitals, Saveetha Institute of Medical and Technical Sciences, Saveetha University, Chennai, IND

**Keywords:** oral care, ethanolic extract, antioxidant, antibacterial, mouthwash, free radicals, isoflavones, red clover

## Abstract

Introduction: Red clover, a perennial herbaceous plant, has been demonstrated to possess blood-purifying, expectorant, and calming properties. This research endeavors to create and evaluate the antimicrobial, antioxidant characteristics, and cytotoxic effects of the ethanolic extract derived from red clover.

Methods: A water-based solution of red clover was formulated and subjected to centrifugation. Various concentrations of the extract were applied to the wells of agar plates inoculated with *E. coli, Staphylococcus aureus, Streptococcus mutans, Enterococcus faecalis, *and* Candida albicans* and then left to incubate. The inhibition zones for each concentration were subsequently measured. The antioxidant properties were evaluated using the 2,2-diphenyl-1-picrylhydrazyl (DPPH) assay, while the cytotoxicity of the extract was assessed through the brine shrimp lethality assay.

Results: Initially, the extract was tested with a volume of 10 μL, which was subsequently incremented to 20 μL, 30 μL, 40 μL, and 50 μL. According to the DPPH assay, as the concentration of the extract solution increased incrementally by 10 μL, its antioxidant activity also exhibited a corresponding rise. The cytotoxicity assay indicated that the mouthwash formulated with red clover had minimal cytotoxic effects within the range of 5-20 µL. Antibacterial analysis revealed a similar zone of inhibition between the test and control groups.

Conclusion: The ethanolic extract obtained from red clover was identified as a powerful antioxidant, antibacterial, and biocompatible substance. Hence, it can be a potential candidate for application as a mouthwash.

## Introduction

Oral hygiene maintenance is essential for preventing the formation of plaque, a sticky bacterial film that builds up on the teeth along with food particles. Practices for oral hygiene encompass mechanical aids like toothbrushes, dental floss, interdental cleansers, as well as chemotherapeutic agents like mouthwashes, toothpaste, and chewing gums. Mouthwashes, also known as mouth rinses, are liquid solutions designed to reduce the microbial load in the mouth [1]. They offer a safe and effective chemical approach to reduce or eliminate plaque buildup, along with aiding in bacteria removal or destruction, alleviating oral tissue infections, preventing dental decay, and masking bad breath, among other benefits. Many commercially available mouthwashes contain alcohol and other chemicals such as chlorhexidine gluconate and triclosan, which can cause various side effects ranging from altered taste to allergic reactions like contact stomatitis [2]. To mitigate these side effects, nontoxic herbal mouthwashes incorporating various herbs and plant extracts have been introduced. Numerous studies have been conducted to demonstrate the efficacy of herbal mouthwashes [3].

Red clover *(Trifolium pratense*) is among the 250 types of *Trifolium *genus plants, part of the Leguminosae family. Its cultivation in Europe dates back to the third or fourth century [4]. Red clover, a perennial herbaceous plant found in temperate and subtropical regions worldwide, is recognized for its medicinal properties. Traditional herbalists have long used it for its roles as a blood purifier, expectorant, alternative medicine, and sedative. Its efficacy in treating various inflammatory conditions such as rheumatism and upper respiratory issues, as well as its mild sedative and antispasmodic effects, have been attributed to its high content of mildly estrogenic isoflavones [5]. Alongside isoflavonoids, *Trifolium pratense* contains saponins, volatile oils, cyanogenic glycosides, coumarin derivatives, flavonoids, and trace amounts of vitamins and minerals, contributing to its antibacterial properties. Consequently, it has been employed to alleviate inflammatory symptoms and promote bone health [6]. Wang and colleagues identified biochanin A in red clover extracts [7]. In 1965, Schultz demonstrated that both biochanin A and formononetin exist as glycosides within red clover [8].

There has been growing interest in natural antioxidants present in plants due to their well-known medicinal and nutritional advantages. A key factor contributing to the beneficial effects of compounds and extracts derived from plants is their antioxidant properties. Ethnopharmacological studies suggest that approximately 80% of plant-based medicines may owe their therapeutic benefits to traditional medicinal uses [9]. Natural antioxidants participate in various metabolic processes, including adjusting intracellular redox potential, inhibiting the generation of reactive oxygen species (ROS), and directly or indirectly neutralizing free radicals [10].

Plant extracts are extensively utilized in the pharmaceutical sector due to their natural availability and reduced incidence of side effects. However, there is currently insufficient literature evidence to substantiate the effectiveness of using red clover isoflavones as a mouthwash for oral hygiene maintenance [11]. Hence, this study aimed at formulating an ethanolic extract of red clover and evaluate its antibacterial and antioxidant properties, as well as assess its cytotoxic effects.

## Materials and methods

Preparation of the extract 

**Figure 1:**
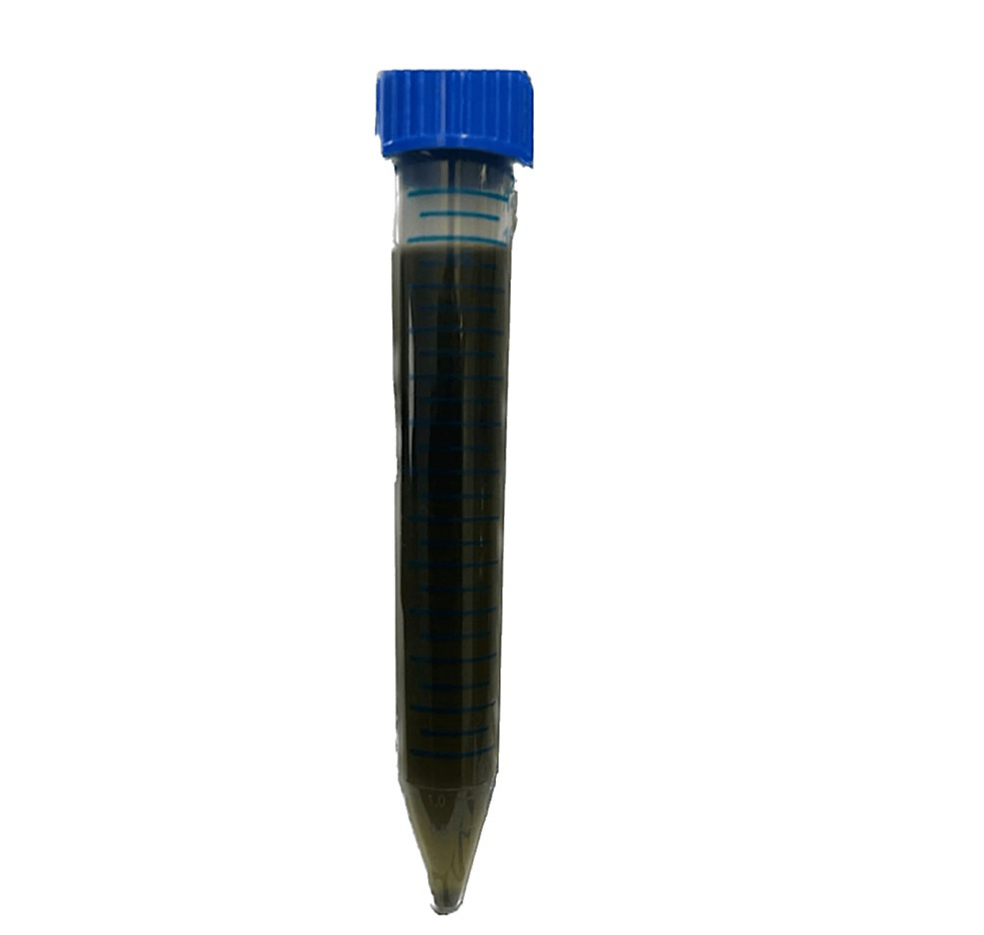
Red clover extract Red clover extract prepared using double filtration method

Dried red clover obtained from stock seed farms in Murdock, USA, was used for the extraction process. Five grams of the dried red clover were placed in a conical flask, and 50 ml of 95% ethanol was added as a solvent to create the crude extract. The mixture was then heated in a microwave for 20 minutes with stirring every five minutes. Afterward, it was allowed to cool in a condenser for 45 minutes. The extract was filtered and cooled to room temperature in a centrifuge, yielding the final extract (Figure 1).

Antibacterial assay 

The agar well diffusion technique was employed to assess the antibacterial activity of the synthesized red clover extract. Various concentrations of the extract (25 μL, 50 μL, 100 μL) were introduced into the prearranged wells, and the plates were then incubated at 37°C for 24 hours to observe its impact on *S. aureus, E. coli, S. mutans, E. faecalis, *and *C. albicans.* Amoxicillin antibiotics were utilized as a positive control. The diameter of the inhibition zone (measured in millimeters) was documented.

Antioxidant activity

The antioxidant activity of the extract was assessed using the 2,2-diphenyl-1-picrylhydrazyl (DPPH) assay. Various concentrations (10 μL, 20 μL, 30 μL, 40 μL, and 50 μL) of the extract were combined with 1 ml of 0.1 mM DPPH in methanol solution and 450 μL of 50 mM Tris HCl buffer (pH 7.4), followed by a 30-minute incubation period. Subsequently, the reduction in the quantity of DPPH free radicals was gauged by measuring the absorbance at 517 nm. Butylated hydroxytoluene (BHT) was utilized as a control. The percentage of inhibition was determined using the following formula: % inhibition = (absorbance of control - absorbance of sample)/absorbance of control x 100

Cytotoxicity assay

The cytotoxic effects of both a commercial mouthwash and a red clover-based mouthwash were evaluated using the brine shrimp method. Brine shrimp eggs (*Artemia salina*) were sourced locally from Aquatic Remedies, Chennai, and were hatched in artificial seawater comprising sea salt at a concentration of 40 g/L. Following a 48-hour incubation period at 22°C-29°C. Pasteur pipette was used to collect the nauplii. Subsequently, 10 nauplii were introduced into each well, which was filled with an NaCl solution. Varying concentrations (ranging from 25 μL, 50 μL, 100 μL) of the extract were added to each well. The wells were then left undisturbed for 24 hours, after which the percentage of live nauplii in each well was counted.

## Results

Antibacterial assay 

Different concentrations of red clover showed significant antibacterial activity against the tested pathogens, as evidenced by the zones of inhibition. The zone of inhibition observed for* E. faecalis and C. albicans* at a concentration of 100 μL was notably larger than that of the control group. However, for the other species, the zone of inhibition was comparable to that of the control group (Table 1).

**Table 1:** Antibacterial analysis Zone of inhibition (in mm) was observed for oral pathogens

Microorganism	Zone of inhibition (in mm) at different concentrations			
	25 μL	50 μL concentrations	100 μL concentrations	Control (amoxicillin)
S. aureus	9	9	11	13
C. albicans	9	9	16	12
E. faecalis	9	15	24	22
S. mutans	9	9	13	14
E. coli	10	10	16	24

Antioxidant activity 

**Figure 2:**
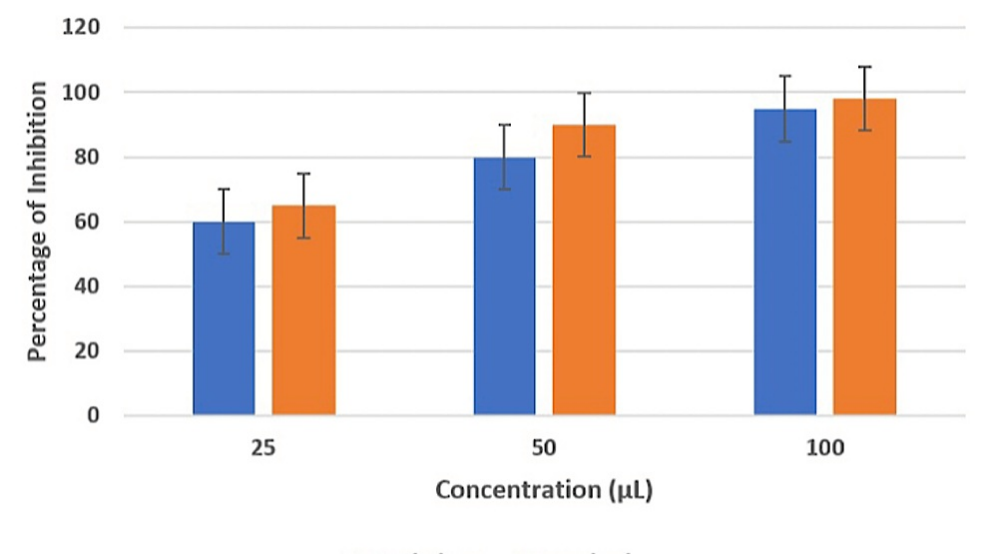
Antioxidant assay DPPH: 2,2-diphenyl-1-picrylhydrazyl DPPH assay was performed to test antioxidant activity

Initially, the extract was tested at a concentration of 10 μL, which was subsequently incremented to 20 μL, 30 μL, 40 μL, and 50 μL. According to the DPPH assay, as the concentration of the extract solution increased by 10 μL with each test, its antioxidant activity also demonstrated a corresponding increase on a scale, and the antioxidant activity of the red clover extract was found to be comparable to that of the control (Figure 2).

Brine shrimp lethality assay

In the cytotoxicity test, the viability of nauplii was assessed using the prepared extract. Results showed that nauplii survived across different concentrations of red clover extract. Notably, at lower concentrations, nauplii showed higher survival rates at both 24 and 48 hours compared to higher concentrations. The survival rate of nauplii fell within acceptable ranges (Table 2).

**Table 2:** Cytotoxicity test Brine shrimp lethality assay was done to test cytotoxicity

Red clover extract concentrations (in µl)	Survival of nauplii (percentage) baseline	Survival of nauplii (percentage) 24 hrs	Survival of nauplii (percentage) 48 hrs
10	100	70	60
20	100	65	60
30	100	60	50
40	100	55	50
50	100	50	40

## Discussion

The primary objective of this investigation was to formulate an ethanolic extract derived from red clover and assess its antioxidant, antibacterial, and cytotoxic attributes. Comparative analysis was conducted between the prepared extract and a commercial mouthwash containing chlorhexidine. The findings revealed that the extract exhibited minimal cytotoxicity compared to the control. Furthermore, antibacterial and antioxidant evaluations demonstrated significant antimicrobial efficacy of the ethanolic red clover extract against various oral pathogens, particularly* E. faecalis *and* C. albicans. *Prior research has extensively explored the beneficial effects of herbal mouthwashes compared to chlorhexidine mouthwash [12-14]. Additionally, Kothari et al. conducted studies on the antimicrobial properties of clove and cinnamon [15].

The antimicrobial assessment of the ethanolic extract derived from red clover revealed significant efficacy against various pathogens. The zone of inhibition was notably highest against *E. faecalis *and* C. albicans*. Conversely, other pathogens displayed comparable outcomes to the control group, namely, amoxicillin. The antimicrobial activity of the red clover extract is attributed to its bioactive constituents such as flavonoids and isoflavones, which disrupt bacterial growth by interfering with their cell membranes and inhibiting essential enzymes and signaling pathways. Previous research has also highlighted the antimicrobial properties of red clover phenolic extract against the ammonia-producing bacterium. These findings suggest that the phenolic compounds present in red clover may play a role in inhibiting amino acid fermentation [16]. The mechanism underlying its antibacterial effect appears to align with previous studies. The antimicrobial properties of red clover can assist in decreasing the bacterial presence in periodontal pockets, thereby supporting the treatment of periodontal infections and halting the advancement of the disease.

The antioxidant potential of the extract was evaluated through the DPPH assay, revealing a correlation between increased concentrations of the extract and enhanced antioxidant activity. As mentioned earlier, red clover comprises flavonoids and phenolic acids, which play a crucial role in neutralizing free radicals within the body, consequently mitigating oxidative stress [17]. Consequently, red clover holds promise in reducing the risk of chronic diseases. Research conducted by Lee et al. and Khorasani et al. has corroborated the presence of phenolic and flavonoid compounds in red clover, underscoring their contribution to these beneficial properties [18-19]. Red clover might aid in tissue regeneration by minimizing oxidative harm to periodontal tissues.

The brine shrimp lethality assay was employed to investigate cytotoxicity, revealing a greater number of live nauplii at lower concentrations (5-20 µL). However, lethality increased as the concentration of the extract rose. Similar to numerous natural substances, the cytotoxic effects of red clover may follow dose-dependent patterns. There exists evidence indicating the potential cytotoxicity of red clover extract against various cell types [20]. This highlights the importance of optimizing the extract's concentration to mitigate its cytotoxic effects.

Limitations 

This study encountered several limitations. Firstly, further examination of the cytotoxic effects of the extract should encompass diverse cell lines, as its impact may vary depending on the cell type. Secondly, an analysis of the antibacterial activity against periodontal pathogens, particularly gram-negative bacteria, is warranted. Lastly, additional investigations involving animal models and clinical settings are necessary to provide a more comprehensive understanding of the extract's potential effects.

## Conclusions

The ethanolic extract obtained from red clover demonstrated satisfactory antimicrobial and antioxidant characteristics, along with minimal cytotoxicity. Consequently, it appears promising as a natural component for oral care products, such as mouthwash, in the fight against periodontal diseases. However, further research in the form of clinical trials is essential to thoroughly assess its effectiveness and safety for this particular application.
